# Focal adhesion alterations in G0‐positive melanoma cells

**DOI:** 10.1002/cam4.5510

**Published:** 2022-12-19

**Authors:** Alexandra R. Esimbekova, Nadezhda V. Palkina, Ivan S. Zinchenko, Vasiliy D. Belenyuk, Andrey A. Savchenko, Ekaterina Yu Sergeeva, Tatiana G. Ruksha

**Affiliations:** ^1^ Department of Pathophysiology Krasnoyarsk State Medical University Krasnoyarsk Russia; ^2^ Laboratory of Cell Molecular Physiology and Pathology Federal Research Center, Krasnoyarsk Science Center of The Siberian Branch of The Russian Academy of Sciences Krasnoyarsk Russia

**Keywords:** cell cycle, focal adhesion, G0, melanoma, quiescence, senescence

## Abstract

**Background:**

Melanoma is a highly heterogeneous malignant tumor that exhibits various forms of drug resistance. Recently, reversal transition of cancer cells to the G_0_ phase of the cell cycle under the influence of therapeutic drugs has been identified as an event associated with tumor dissemination. In the present study, we investigated the ability of chemotherapeutic agent dacarbazine to induce a transition of melanoma cells to the G_0_ phase as a mechanism of chemoresistance.

**Methods:**

We used the flow cytometry to analyze cell distribution within cell cycle phases after dacarbazine treatment as well as to identifyG_0_‐positive cells population. Transcriptome profiling was provided to determine genes associated with dacarbazine resistance*.* We evaluated the activity of β‐galactosidase in cells treated with dacarbazine by substrate hydrolysis. Cell adhesion strength was measured by centrifugal assay application with subsequent staining of adhesive cells with Ki‐67 monoclonal antibodies. Ability of melanoma cells to metabolize dacarbazine was determined by expressional analysis of CYP1A1, CYP1A2, CYP2E1 followed by CYP1A1 protein level evaluation by the ELISA method.

**Results:**

The present study determined that dacarbazine treatment of melanoma cells could induce an increase in the percentage of cells in G_0_ phase without alterations of β‐galactosidase positive cells which corresponded to the fraction of the senescent cells. Transcriptomic profiling of cells under dacarbazine induction of G_0_‐positive cells percentage revealed that ‘VEGFA‐VEGFR2 signaling pathway’ and ‘Cell cycle’ signaling were mostly enriched by dysregulated genes. ‘Focal adhesion’ signaling was also found to be triggered by dacarbazine. In melanoma cells treated with dacarbazine, an increase in G_0_‐positive cells among adherent cells was found.

**Conclusions:**

Dacarbazine induces the alteration in a percentage of melanoma cells residing in G_0_ phase of a cell cycle. The altered adhesive phenotype of cancer cells under transition in the G_0_ phase may refer to a specific intercellular communication pattern of quiescent/senescent cancer cells.

## Graphical abstract

The present study revealed that the alkylating agent dacarbazine induces an increase in G_0_‐positive melanoma cells that correspond to quiescent and senescent cells. Further transcriptional experiments showed that this was combined with senescence‐associated secretory phenotype transcriptional pattern and focal adhesion signaling alteration. Indeed, G_0_‐positive cells exhibited increased adhesive capacities. A specific adhesive phenotype of G_0_‐positive cells may be involved in intercellular communication as a part of tumor dissemination.

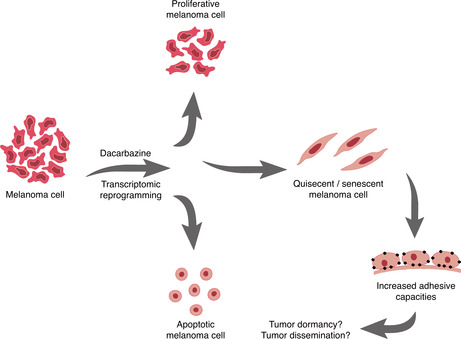

## 1 INTRODUCTION

Melanoma is a skin cancer with a high rate of cancer‐associated mortality, which is partly due to the tumor's high resistance to anticancer treatment.^1^ The treatment paradigm for melanoma changed in the last decade with targeted and immunotherapy presentation.^2^ However, despite the recent progress in melanoma therapeutics, patients with advanced melanoma have a limited overall survival rate.^3^ The tumor is characterized by developing multidrug resistance that corresponds to high plasticity and heterogeneity of melanoma cells. The application of single cell analysis allowed to highlight several cell‐specific transcription programs and phenotypes associated with the progression and dissemination of the tumor. Thus, MITF transcription factor is considered to be a master regulator of proliferative phenotype of melanoma cells whereas transforming growth factor β, tumor necrosis factor α and WNT5A facilitate transition to invasive phenotype.^4^ Recent studies highlighted the population of slow cycling/quiescent cancer cells as resistant to classical chemotherapy.^5^ Subpopulation of melanoma slow cycling cells was not related to BRAF, NRAS, and PTEN mutation status but was characterized by increased invasive potential.^6^ Meanwhile, transcription factor SOX10 diminished expression leads to acquisition of low proliferative phenotype that is associated with resistance of BRAF‐mutant melanoma cells to BRAF inhibitors.^7^ The alkylating agent dacarbazine was introduced for melanoma therapy more than four decades ago,^8^ but soon thereafter data concerning its limited clinical efficacy were reported.^9^ However, dacarbazine remains the chemotherapeutic drug for disseminated forms of a tumor, and is used in combination anticancer therapy as well as a reference agent for novel treatment options in metastatic melanoma.^10^ Dacarbazine causes DNA methylation by transferring methyl adducts to DNA bases, which induces DNA repair processes, including DNA mismatch repair and base‐excision repair by poly (ADP‐ribose) polymerase activation.^11^ The mode of cancer cell death induced by DNA damage triggered by alkylating agents is unpredictable and can occur as apoptosis or necrosis where p53‐independent apoptosis is the prevalent event.^12^ The enzyme O6‐methylguanine‐DNA methyltransferase (MGMT) reverses DNA injury; thus, its activity is associated with the inefficiency of alkylating drugs.^13^ Dacarbazine is characterized by a low response rate that does not exceed 12%, although the mechanisms responsible for its limited efficacy have not been established.^2^ Recent studies on tumor resistance highlighted the impact of quiescent/senescent cancer cells in this process.^14^ Quiescent cells represent a population of slow‐cycling or non‐proliferating cells that reside within a tumor in the G_0_ phase of the cell cycle. Quiescent cells can reversely enter the cell cycle, re‐proliferate and lead to a new phase of tumor growth.^15^ Therefore, disseminated tumor cells are considered the basis for minimal residual disease via the acquisition of quiescence, thus retaining their ability for tumor relapse.^16^ By contrast, senescent cells, which are also G_0_ positive, are canonically characterized by irreversible cell cycle arrest.^17^ Quiescent cells are characterized by their diminished metabolic activity, RNA content and protein synthesis, whereas senescent cells exhibit increased levels of metabolic processes. Both G_0_‐positive cell types are characterized by triggering p53, and the CDK inhibitors p21 and p27.^18^, ^19^ Senescent cells produce the senescence‐associated secretory phenotype (SASP), which is represented by IL‐1, IL‐6 and IL‐8 release, and is implicated in tumor microenvironment remodeling promoting a pro‐inflammatory phenotype,^20^ while quiescent cells are evidently implicated in cancer resistance by evading apoptosis‐triggered stimuli induced by anticancer agents, thus escaping the immune response.^21^ However, recent studies provide more evidence that senescence might be reversible in cancer.

Dacarbazine and other alkylating agents exhibited an ability to induce both G_1_ and G_2_ arrest, although G_2_/M‐arrested cells were more susceptible to apoptosis induction, opposite to more resistant G_1_‐arrested cells, which may reversibly exit from the cell cycle or develop endoplasmic reticulum stress, thus enhancing the degradation of damaged proteins.^22^ Endoplasmic reticulum stress results in a switch of melanoma cells from proapoptotic to antiapoptotic signaling under stressful stimuli.^23^ Cancer cell resistance was shown to correlate with proliferative state of a cell. Transcriptomic profiling revealed heterogeneous phenotype of resistant cancer cells but highly associated with slow cycling.^24^

Thus, the G_0_ transition is considered to be a mechanism by which cancer cells can acquire mutations and provide the transcriptional reprogramming necessary for further tumor progression following the action of various stressful factors, including anticancer agents.^25^ Since exit from the cell cycle can impact the resistanceof cancer cells to dacarbazine, the aim of the present study was to determine if dacarbazine affected the percentage of G_0_‐positive cells in melanoma.

## 2 METHODS

### 2.1 Cell lines and culture conditions

The cell lines used in the present study were human melanoma SK‐MEL‐2 (cat. no. ATCC® HTB‐68™) and human melanoma BRO, which were obtained as a gift from *Federal State Budgetary Scientific Institution Research Institute of Fundamental and Clinical Immunology*. The cells were cultured in DMEM (cat. no. С470п; PanEko) supplemented with 10% fetal bovine serum (FBS) (HyClone; Cytiva), 1% antibiotic/antimycotic (cat. no. 15240062; Gibco; Thermo Fisher Scientific, Inc.) and maintained in an incubator at 37°C with 5% CO_2_ (Sanyo MSO‐5 AC; Sanyo Electric Co., Ltd.).

### 2.2 Flow cytometric analysis of cell cycle with propidium iodide DNA staining

Сells were cultured in a 25‐cm^2^ flask and treated with dacarbazine for 72 h at 37°C at a concentration of 1.2 or 2.4 mM. After 72 h, the cells were washed with PBS, and fresh medium without the aforementioned compound was added and incubated for a further 48 h to allow the transition of cells into G_0_, which happens within 5 days after incubation with a chemotherapeutic drug in accordance with the literature, whereas apoptotic cells should be eliminated.^26^, ^27^ DMSO‐treated cells were used as a control. Next, the cell suspensions were washed with PBS, fixed with 70% ice‐cold ethanol, treated with RNase A (100 μg/ml) (Invitrogen; Thermo Fisher Scientific, Inc.) for 30 min, stained with propidium iodide (PI; 100 μg/ml) for 30 min at 37°C in the dark and subjected to flow cytometry using a Cytomics FC‐500 flow cytometer (Beckman Coulter, Inc.). The experiments were performed three times.

### 2.3 Ki‐67 and flow cytometry‐based cell cycle analysis

The effects on the cell cycle phase distribution of the melanoma cells were assessed by flow cytometry. Each melanoma cell line was cultured in a 25‐cm^2^ flask and treated with dacarbazine (IC_50_ and 2‐fold IC_50_) for 72 h at 37°C. Subsequently, the cells were washed with PBS (Helicon), replaced in fresh medium without the compound and incubated for a further 48 h. Next, the cell suspensions were washed with PBS, fixed with 10% formalin, permeabilizated with 0.1% Triton X‐100 (GERBU Biotechnik GmbH), and stained with an anti‐Ki‐67 monoclonal antibody conjugated to Fluorescein (FITC; cat. no. SolA15; eBioscience; Thermo Fisher Scientific, Inc.) at a concentration of 1:100 within 4 h at room temperature and 100 μg/ml PI staining solution (Invitrogen; Thermo Fisher Scientific, Inc.) for 20 min at room temperature. The proportion of cells in each phase was detected using a Cytomics FC‐500 flow cytometer (Beckman Coulter, Inc.) using CXP software (version 2.2; Beckman Coulter, Inc.). Gating strategies for determining the G0 cell population were taken from the cell state analysis protocol^28^ and based on differences in the level of expression of the Ki‐67 marker, as well as the content of RNA in them, which was characterized by PI staining. Generally, the G0 cells have lower levels of Ki‐67 and RNA levels, so these cells may be distinguishable from other proliferating cells. Gating was carried out in the range of values ≤10^0^ of Ki‐67‐FITC fluorescence (negative), and within a PI fluorescence range between 0.7 and 1.3 relative units. The experiment was performed in triplicate.

### 2.4 Identification of β‐galactosidase‐positive cells

To assess the levels of dacarbazine‐induced senescent melanoma cells, the expression of senescence‐associated enzyme β‐galactosidase was determined. Briefly, SK‐MEL‐2 and BRO melanoma cells were seeded in 24‐well plates and treated with dacarbazine as aforementioned. At the end of the treatment, the cells were washed with PBS and fixed with a 4% formaldehyde solution for 20 min at room temperature. The cells were then again washed with PBS and stained with a freshly prepared staining solution for 2 h in the dark on a thermostat at 37°C. The staining solution contained 1X citric acid/sodium phosphate buffer (pH 6.0) (cat. no. ab64214; Abcam) 5 mM potassium ferricyanide (Reakhim), 5 mM potassium ferrocyanide (Reakhim), 150 mM NaCl (Reakhim), 2 mM MgCl_2_ (Reakhim) and 1 mg/ml 5‐bromo‐4‐chloro‐3‐indolyl‐β‐D‐galactopyranoside (Invitrogen; Thermo Fisher Scientific, Inc.), which was hydrolyzed under the catalysis of senescent cell β‐galactosidase to form a blue product that could be visualized under a microscope. Finally, the cells were visualized using an inverted microscope (MIB‐R; LOMO‐Microsystems), photographed and analyzed in ≥10 fields of view. The total number of cells in the visual fields and the number of cells colored in blue were counted. The level of cell aging was calculated as the percentage of stained cells in each sample. The experiment was repeated three times.

### 2.5 Immunocytochemistry

Cells were cultured in 24‐well plates, washed with PBS, fixed with 10% formalin and permeabilized with 0.5% Triton X‐100 for 10 min at room temperature. Next, the cells were incubated with a primary rabbit monoclonal antibody against human Ki‐67 (cat. no. ab15580; Abcam; dilution 1:100) with 10% FBS at 4°C overnight. Goat anti‐rabbit Alexa Fluor 488 IgG (H + L) antibody was used as a secondary antibody at 1:200 dilution (cat. no. A‐11008; Invitrogen; Thermo Fisher Scientific, Inc.) for 1 h at room temperature in the dark. The cell nuclei were counterstained with 1 μg/ml DAPI (AppliChem GmbH) for 15 min at room temperature. The cells were counted in ≥10 visual fields using the FLoid™ Cell Imaging Station (Thermo Fisher Scientific, Inc.). The nuclei of proliferating cells were stained green and blue, while the nuclei of non‐proliferating live cells were stained blue. The results were expressed as the mean of the percentage of cells in G_0_ stage.

### 2.6 Microarray

Isolation of total RNA was performed using the RecoverAll™ Total Nucleic Acid Isolation kit (Ambion; Thermo Fisher Scientific, Inc.) according to the manufacturer's protocol. For whole transcriptome analysis, samples containing total RNA were diluted to a concentration of 250 ng/μl. Synthesis of a single cDNA strand was performed in a thermocycler for 1 h at 25°C, followed by 1 h at 42°C and 2 min at 4°C using First‐Strand Buffer and First‐Strand Enzyme reagents (Applied Biosystems; Thermo Fisher Scientific, Inc.). Poly‐A RNA Control Stock diluted with Poly‐A RNA Control Dil Buffer from the GeneChip™ Poly‐A RNA Control Kit (Applied Biosystems; Thermo Fisher Scientific, Inc.) was added as an exogenous positive control. Next, the resulting single‐stranded cDNA was converted to double‐stranded cDNA by adding Second‐Strand Buffer and Second‐Strand Enzyme (Applied Biosystems; Thermo Fisher Scientific, Inc.) and incubating in a thermocycler for 1 h at 16°C, followed by 10 min at 65°C and 2 min at 4°C. Next, сomplementary DNA (cDNA) synthesis and amplification were performed on a double‐stranded DNA template at room temperature using in vitro transcription (IVT) Buffer and IVT‐Enzyme (Applied Biosystems; Thermo Fisher Scientific, Inc.) and incubation in a thermocycler for 16 h at 40°C. The amplified cRNA was purified using magnetic Purification Beads (Applied Biosystems; Thermo Fisher Scientific, Inc.) on a magnetic plate, followed by a three‐step wash with 80% ethanol, and eluted with nuclease‐free water heated to 65°C. The RNA concentration in the eluates was adjusted to 625 ng/μl with nuclease‐free water and converted to DNA by reverse transcription (RT) in a thermal cycler for 5 min at 70°C, 5 min at 25°C and 2 min at 4°C using reagents 2nd‐Cycle Primers, 2nd‐Cycle ss‐cDNA Buffer and 2nd‐Cycle ss‐cDNA Enzyme (Applied Biosystems; Thermo Fisher Scientific, Inc.). The resulting cDNA was hydrolyzed with RNase H (Applied Biosystems; Thermo Fisher Scientific, Inc.) by incubation in a thermocycler as follows: 45 min at 37°C, 5 min at 95°C and 2 min at 4°C. Upon hydrolysis, cycle 2 single‐stranded cDNA was also purified using magnetic particles with a three‐step wash with 80% ethanol. Purified cDNA was fragmented using 10X cDNA Fragmentation Buffer, Uracil‐DNA glycosylase (UDG) (10 U/μl), apurinic/apyrimidinic endonuclease 1 (1000 U/μl) from the GeneChip™ WT Terminal Labeling Kit (Applied Biosystems; Thermo Fisher Scientific, Inc.) in a thermal cycler for 1 h at 37°C, followed by 2 min at 93°C and 2 min at 4°C, and labeled with 5X TdT Buffer, 5 mM DNA Labeling Reagent and 30 U/μl TdT from the above GeneChip™ WT Terminal Labeling Kit by incubating in a thermal cycler for 1 h at 37°C and 10 min at 70°C. Upon labeling, the samples were subjected to microarray hybridization reaction using 5X WT Hyb Add 1 reagents (Affymetrix; Thermo Fisher Scientific, Inc.), 3 nM Control Oligo™ B2 and 20X Hybridization Controls (bioB, bioC, bioD and cre) (Applied Biosystems; Thermo Fisher Scientific, Inc.), as well as 1.5X WT Hyb Add 4 and 2.5X WT Hyb Add 6 (Affymetrix; Thermo Fisher Scientific, Inc.) in the module of the GeneAtlas™ Hybridization Station (Affymetrix; Thermo Fisher Scientific, Inc.) for 20 h at 48°C. Upon hybridization, the microarrays were washed in a Fluidic Station module (Affymetrix; Thermo Fisher Scientific, Inc.). The intensity of the fluorescent signal with conversion to digital data was detected using an Imaging Station module (GeneAtlas® Microarray System; Affymetrix; Thermo Fisher Scientific, Inc.). The selection of differentially expressed transcripts was based on *p* < 0.05 statistical significance using a false detection rate and a fold‐change value of ≥2. The data were deposited in ArrayExpress Archive E‐MTAB‐11399 (https://www.ebi.ac.uk/arrayexpress/experiments/E‐MTAB‐11399).

### 2.7 Bioinfomatical analysis

We used theTranscriptome Analysis Console Software v.4.0.1. (Thermo Fisher Scientific, Inc.) to analyze gene expression patterns of melanoma cells after dacarbazine treatment. The expression data were also used to cluster the samples using a hierarchical clustering method. All *p*‐values were false discovery rate‐corrected for multiple hypothesis testing. Differentially expressed probe sets were defined using the threshold of absolute fold change ≥2 and the *Q*‐value ≤0.05.

### 2.8 Quantitative PCR (qPCR)

Isolation of total RNA was performed using the RecoverAll™ Total Nucleic Acid Isolation kit (Ambion; Thermo Fisher Scientific, Inc.) according to the manufacturer's protocol.Next, the isolated RNA was converted into cDNA using the random primers, moloney murine leukemia virus (MMLV) reverse transcriptaseand buffers included in the MMLV RT kit (Eurogen). Each sample consisted of 10 μl cDNA, 6 μl template RNA, 3 μl primers and 11 μl reaction mixture (consisting of 4 μl 5X buffer for the synthesis of the first strand of cDNA, 2 μl dNTP mix, 2 μl DTT, 1 μl MMLV reverse transcriptase and 2 μl RNase‐free H_2_O). RT was carried out at a temperature of 40°С for 50 min, and was subsequently stopped by heating the samples at 70°С for 10 min. Next, the resulting cDNA was amplified on the StepOne™ Real‐Time PCR System (Applied Biosystems; Thermo Fisher Scientific, Inc.). The reaction mixture contained 20 μl per sample, consisting of 2 μl cDNA, 9 μl deionized water, 8 μl 2.5X reaction mixture for qPCR in the presence of a reference dye carboxyrhodamine (ROX) (Synthol) and 1 μl 20X primer solution from the following kits for determining the levels of microRNA expressionfrom the following gene expression kits: cytochrome P450 family 1 subfamily A member 1 (CYP1A1) Hs01054796_g1, CYP1A2 Hs00167927_ml, CYP2E1 Hs00559367_ml, TP53 Hs01034249_m1, retinoblastoma‐like protein 1 (RBL1) Hs00765700_m1, MGMT Hs01037698_m1, CDK4 Hs01565685_m1 and aurora kinase A (AURKA) Hs01582072_m1 (cat. no. 4331182; Applied Biosystems; Thermo Fisher Scientific, Inc.). The thermocycling protocol was 50°C for 2 min and 95°C for 10 min, followed by 40 cycles of denaturation at 95°С for 15 s, and annealing and elongation at 60°С for 1 min. As endogenous controls to normalize the expression levels of the samples, the levels of glyceraldehyde‐3‐phosphate dehydrogenase (GAPDH)Hs99999905_m1 and hypoxanthine phosphoribosyltransferase 1 (HPRT1)Hs01003267_m1 (cat. no. 4331182; Applied Biosystems, Thermo Fisher Scientific Inc.)^29^ were determined simultaneously using the 2^−ΔΔCq^ method.^30^ Data were obtained from three independent experiments.

### 2.9 Identification of CYP1A1 levels by ELISA

To prepare cell lysates, each sample, consisting of 5 × 10^6^ melanoma cells, was resuspended in 500 μl lysis buffer containing 180 μl distilled water, 130 μl 0.5 М Tris–HCl (pH 6.8) (Reakhim), 420 μl glycerol (Samaramedprom), 210 μl 10% SDS (Thermo Fisher Scientific, Inc.) and 50 μl 2β‐mercaptoethanol (Reakhim), and then centrifuged at 1500*g* for 10 min to remove cell debris. Detection of CYP1A1 in the cell lysates was then conducted with an ELISA kit (cat. no.SED295Hu; Cloud Clone Corp.) at a temperature of 37°С according to the manufacturer's protocol. Briefly, samples, in a volume of 100 μl were added to plates pre‐seeded with antibodies against CYP1A1 for 1 h at room temperature. Reagent A and Reagent B, which are HRP‐conjugated avidin reagents, were then sequentially added to each well and incubated at 37°C for 1 h and 30 min, respectively. The wells were then thoroughly washed, and 3,3′,5,5′‐Tetramethylbenzidine (TMB) substrate was added, causing the wells that contained CYP1A1, biotin‐conjugated antibodies and enzyme‐conjugated avidin to change color. The enzyme‐substrate reaction was terminated by adding a stop solution of sulfuric acid. The absorbance of the resulting complex was measured spectrophotometrically at a wavelength of 450 nm using an Efos‐9305 spectrophotometer (Shvabe Photosystems). The concentration of CYP1A1 in the samples was determined by comparing the optical density of the studied samples to that of the standard curve. The experiment was performed in triplicate.

### 2.10 Cell adhesion

Cells were removed from the culture flasks with a 0.25% trypsin–EDTA solution (Gibco; Thermo Fisher Scientific, Inc.) according to standard procedures. The resulting cell suspension was added to 25‐cm^2^ culture flasks at a concentration of 2 × 10^5^ cells. The cells were incubated in a CO_2_ incubator for 24 h. Cell adhesion was evaluated by centrifugation assay.^31^ Subsequently, the culture flasks were filled with PBS and sealed with a lid. Next, the flasks containing the cells under study were subjected to centrifugal force by centrifugation upwards with a monolayer for 3 min at 1000 rpm. Cells were then washed with PBS, fixed with 10% formalin solution and permeabilized with 0.5% Triton X‐100 solution (Biotechnik GmbH). The fixed cells were stained with a primary rabbit monoclonal antibody against human Ki‐67 (cat. no. ab15580; Abcam; 1:100) supplemented with 10% FBS (HyClone; Cytiva) at 4°C overnight and a secondary IgG goat antibody against rabbit antigens conjugated with Alexa Fluor 488 (H + L) (cat. no. A‐11008; Invitrogen; Thermo Fisher Scientific, Inc.) at a 1:100 dilution at room temperature for 90 min in the dark. The nuclei were stained with a 1:10,000 dilution of DAPI (PanReac AppliChem GmbH) for 15 min at room temperature. On the cell visualizer FLoid™ Cell Imaging Station (Thermo Fisher Scientific, Inc.), the mean number of cells in 5 fields of view in each sample was calculated. Proliferating cells were stained blue and green, while non‐proliferating, G_0_‐positive cells were stained blue only. The experiment was repeated three times.

### 2.11 Statistical analysis

All the experimental procedures were performed in triplicate. Data are presented as the mean ± SEM. Statistical analysis was performed with Mann–Whitney U‐test by using Statistica 7.0 (StatSoft, IncStatistics tool GraphPad Prism (v. 8; GraphPad Software, Inc.; https://www.graphpad.com/)) was used for plotting charts. *p* < 0.05 was considered to indicate a statistically significant difference.

## 3 RESULTS

### 3.1 Expression of the cytochromes CYP1A1, CYP1A2 and CYP2E1 in BRO and SK‐MEL‐2 melanoma cells

**FIGURE 1:**
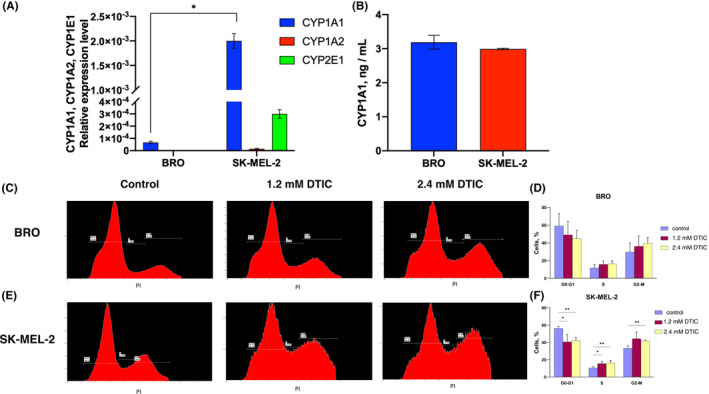
Dacarbazine converting enzymes and the effect of dacarbazine on the cell cycle in melanoma cells. (A) CYP1A1, CYP1A2 and CYP2E1 expression in BRO and SK‐MEL‐2 cells. (B) CYP1A1 protein levels in BRO and SK‐MEL‐2 cells. (C, E) Cells were treated with dacarbazine for 72 h, incubated for an additional 48 h, and then stained with propidium iodide and evaluated by flow cytometry, as shown in the histograms. The left column represents the cell cycle distribution in the control group, while the middle and right columns correspond to BRO and SK‐MEL‐2 cells treated with 1.2 and 2.4 mmol dacarbazine, respectively. (D) BRO melanoma cells showed no alteration in cell cycle distribution upon dacarbazine treatment. (F) SK‐MEL‐2 cells exhibited a decrease in the percentage of cells in G_0_/G_1_ followed by an increase in the percentage of cells in the S and G_2_/M phases of the cell cycle. The figure shows the mean percentage of cells ±the standard error of the mean(Mann–Whitney U test for unpaired samples) was used for statistical analysis (*n* = 3; *p < 0.05 in cells treated with 1.2 mmol dacarbazine vs. control; **p < 0.05 in cells treated with 2.4 mmol dacarbazine vs. control).

SK‐MEL‐2 and BRO melanoma cells were treated with dacarbazine at 1.2 and 2.4 mmol to obtain G_0_‐positive cells, as reported previously, to induce an increase in the percentage of cells in the G_0_ phase of the cell cycle.^32^ Dacarbazine belongs to a family of bioconvertible substances that are mainly metabolized to the N‐demethylated species 5‐(3‐hydroxymethyl‐3‐methyl‐triazen‐1‐yl)‐imidazole‐4‐carboxamide and 5‐(3‐methyl‐triazen‐1‐yl)‐imidazole‐4‐carboxamide by cytochromes P450 in human hepatic cells.^33^ However, extrahepatic dacarbazine metabolism has been reported, as well as extrahepatic expression of dacarbazine converting enzymes.^34^, ^35^ The present study determined the mRNA levels of the cytochromes CYP1A1, CYP1A2 and CYP2E1, as well as the protein levels of cytochrome CYP1A1 in melanoma cells. Both BRO and SK‐MEL‐2 cells expressed cytochrome CYP1A1 at the mRNA and protein levels (Figure 1A,B). In addition, mRNA expression of the cytochromes CYP1A2 and CYP2E1 was identified in SK‐MEL‐2 cells (Figure 1A). These data showed that melanoma cells could convert dacarbazine into its active forms.

### 3.2 Cell cycle phase distribution in melanoma cells following dacarbazine treatment

To determine how cells were distributed in the G_0_/G_1_, S and G_2_/M phases of the cell cycle in accordance with PI incorporation, cells were treated with 1.2 and 2.4 mmol dacarbazine. BRO melanoma cells did not show any alteration in cell cycle phases distribution (Figure 1C,D). By contrast, the percentage of G_0_/G_1_ cells decreased from 56 to 40% (*p* < 0.05), followed by a slight increase in the percentage of cells in the S phase of the cell cycle from 11 to 16% (*p* < 0.05) in SK‐MEL‐2 cells upon treatment with 1.2 mmol dacarbazine. A similar alteration pattern was observed in SK‐MEL‐2 melanoma cells treated with 2.4 mmol of dacarbazine, where the percentage of cells in G_0_/G_1_ phase was reduced to 42% (*p* < 0.05), followed by an increase in the percentage of cells in the S phase of the cell cycle (≤16%; *p* < 0.05). Dacarbazine in a 2.4mmolinduced an increase in the percentage of cells in G_2_/M from 33 up to 42% (Figure 1E,F).

### 3.3 Flow cytometry and immunocytochemistry for the identification of G_0_ ‐positive cells

**FIGURE 2:**
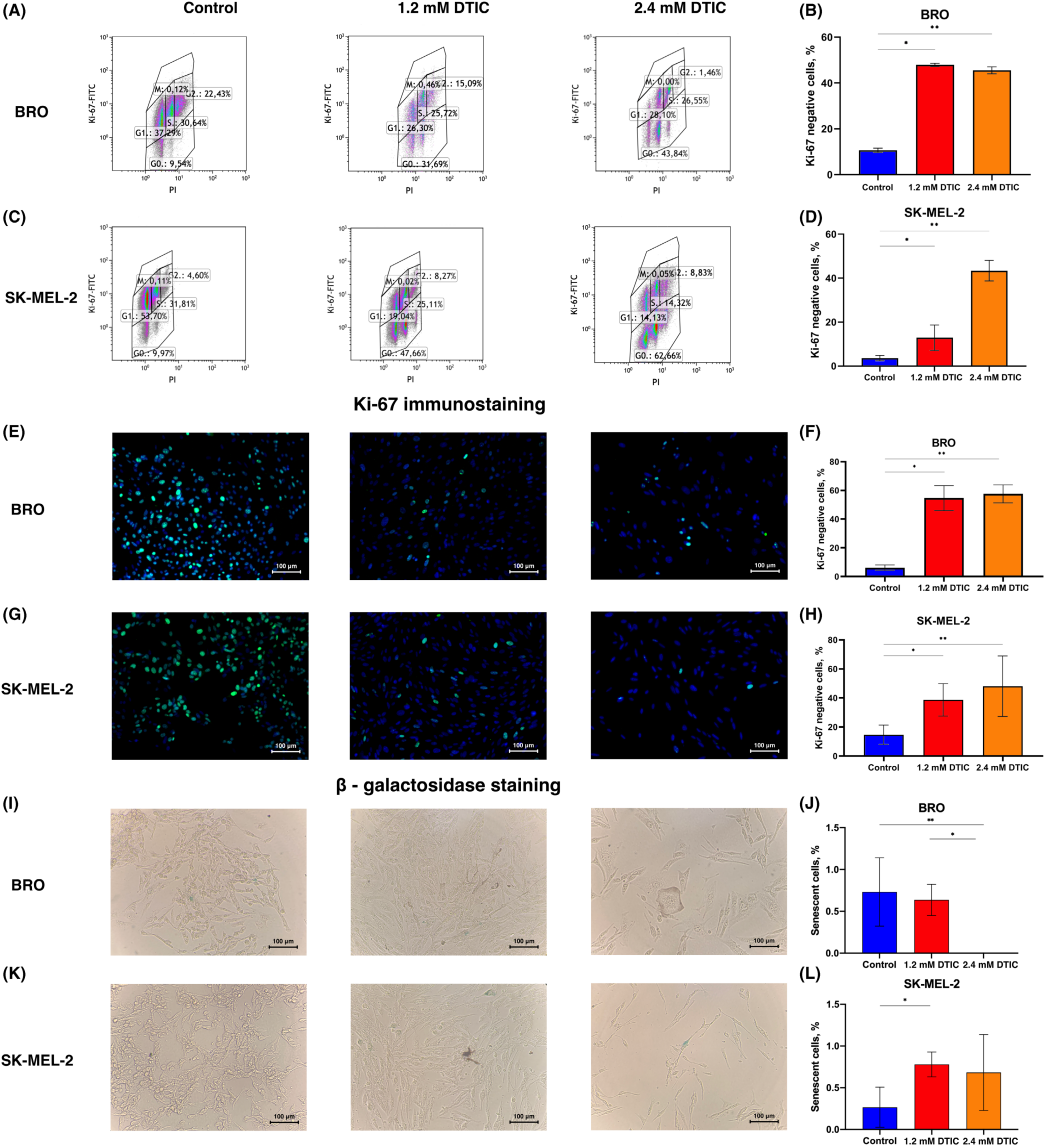
Ki‐67‐negative cells and β‐galactosidase expression levels following dacarbazine treatment at a concentration of 1.2 and 2.4 mmol. Representative flow cytometry dot plots showing the expression of Ki‐67 in propidium iodide‐stained cells. (A, C) Graphs showing the mean ± standard error of the mean(Mann–Whitney U test for unpaired samples) of % Ki‐67‐negative stained cells in (B) BRO and (D) SK‐MEL‐2 cells. An increase in G_0_‐positive cells was found in both cell populations. (E) Dacarbazine at 1.2 and 2.4 mmol increased the percentage of Ki‐67‐negative BRO (E, F) and SK‐MEL‐2 cells (G, H) in accordance to immunocytochemistry. Dacarbazine at 1.2 and 2.4 mmol reduced the percentage of β‐galactosidase‐positive cells in BRO cells (I, J), while 1.2 mmol dacarbazine increased the percentage of β‐galactosidase‐positive SK‐MEL‐2 cells (K, L). Graphs showing β‐galactosidase‐positive stained cells, which corresponded to senescent cells in the control and the 1.2 and 2.4 mmol dacarbazine‐treated (I) BRO and (K) SK‐MEL‐2 cells. The figure shows the mean percentage of cells ±the standard error of the mean. Mann–Whitney U test for unpaired sampleswas used for statistical analysis (*n* = 3; *p < 0.05 in a group treated with 1.2 mmol dacarbazine vs. control; **p < 0.05 in a group treated with 2.4 mmol dacarbazine vs. control).

Staining with monoclonal antibodies against Ki‐67 and PI was used to identify the alterations in the percentage of G_0_‐positive cells by flow cytometry. BRO melanoma cells showed a 4.3‐fold increase in the percentage of G_0_‐positive cells upon dacarbazine treatment at a dose corresponding to 1.2 mmol and 4.0‐fold increase upon 2.4 mmol dacarbazine treatment (Figure 2A,B). In SK‐MEL‐2 cells, 1.2 mmol dacarbazine induced a 4‐fold increase in the percentage of G_0_‐positive cells, while 2.4 mmol led to a 13‐fold increase from 3 to 13 and 43%, respectively (Figure 2C,D).

Ki‐67 immunostaining of melanoma cells upon dacarbazine treatment was performed by immunocytochemistry. The percentage of Ki‐67‐negative cells differed from that of the control 9‐fold, as 6% Ki‐67 negative cells were found in the control, 52% in 1.2 mmol dacarbazine‐treated BRO cells and 58% in 2.4 mmol dacarbazine‐treated BRO cells (Figure 2E,F). The percentage of Ki‐67‐negative cells was higher in dacarbazine‐treated SK‐MEL‐2 cells than in control cells: 37% in cells treated with 1.2 mmol dacarbazine, 48% in cells treated with 2.4 mmol dacarbazine and 15% in the control group (Figure 2G,H).

Cells were incubated with5‐bromo‐4‐chloro‐3‐indolyl‐β‐D‐galactopyranoside, which is a substrate of β‐galactosidase. β‐galactosidase‐positive cells, which are referred to as senescent cells,^36^ were visualized as stained blue. The percentage of β‐galactosidase‐positive BRO and SK‐MEL‐2 cells did not exceed 1% in the control groups. The level of senescent cells was increased in SK‐MEL‐2 cells upon 1.2 mM dacarbazine treatment, from 0.3% to 0.7% (Figure 2K,L), whereas the percentage of senescent cells in BRO cells was not altered following 1.2 mM dacarbazine treatment, but it decreased to 0% upon incubation with 2.4 mM dacarbazine (Figure 2I,G).

### 3.4 Transcriptomic study of SK‐MEL‐2 and BRO cells upon treatment with dacarbazine

In order to understand gene expression alterations corresponded to dacarbazine effects on melanoma cells transcriptome profiling was performed via microarray.

**FIGURE 3:**
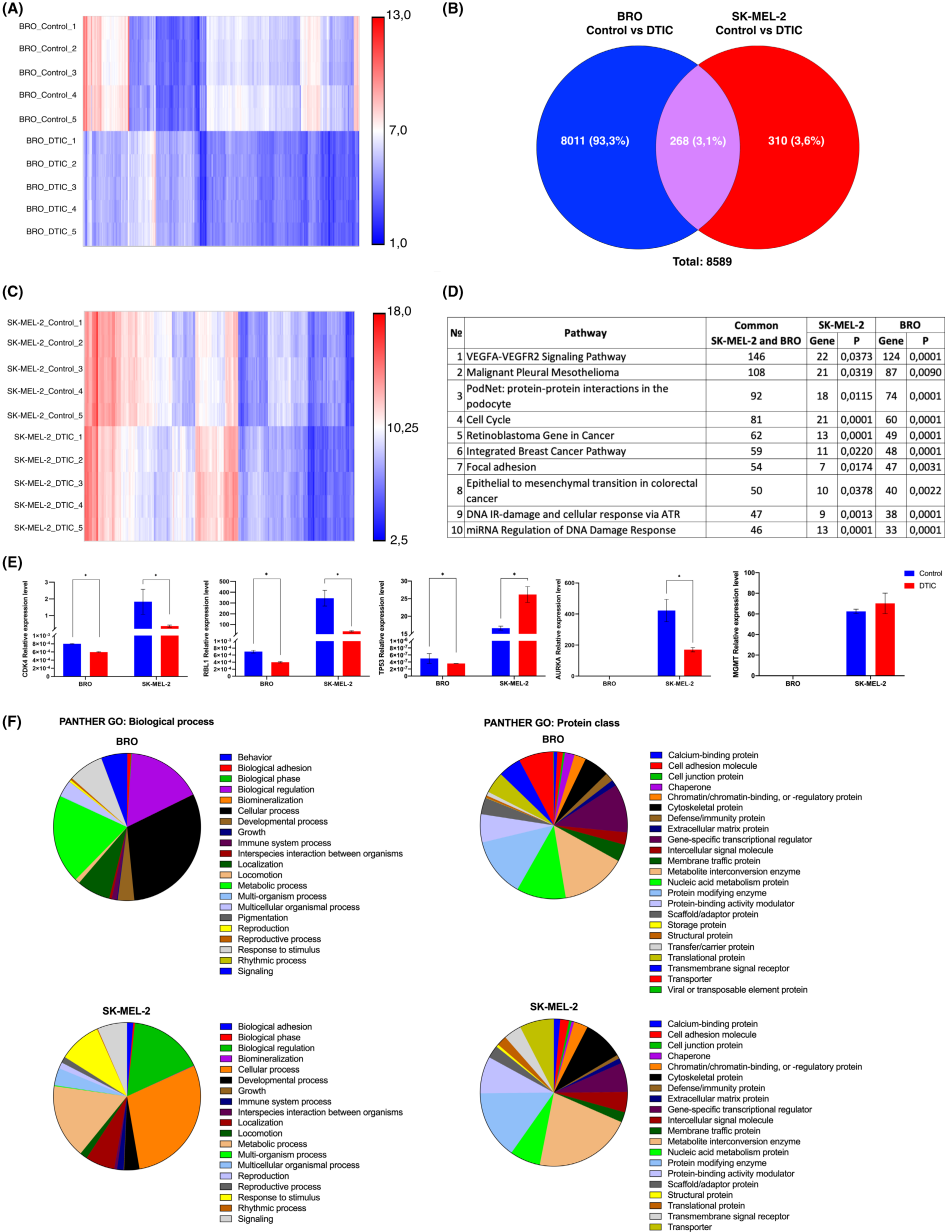
Results of whole transcriptome analysis of BRO and SK‐MEL‐2 melanoma cells. Heatmaps showing differentially altered transcripts in (A) BRO and (C) SK‐MEL‐2 melanoma cells following 1.2 mmol dacarbazine treatment. (B) Venn diagram showing the total number of altered transcripts in BRO and SK‐MEL‐2 melanoma cells upon treatment with 1.2 mmol dacarbazine. (D) Significantly enriched pathways of differentially expressed genes in dacarbazine‐treated BRO and SK‐MEL‐2 melanoma cells versus control. (E) Changes in relative *CDK4*, *RBL1*, *TP53, AURKA* and *MGMT* gene expression levels according to reverse transcription‐quantitative PCR in BRO and SK‐MEL‐2 melanoma cells following 1.2 mmol dacarbazine treatment. Data are presented as the mean ± standard error of the mean. *p < 0.05 by Mann–Whitney U test for unpaired samples. (F) Gene Ontology annotations representing the biological process and protein class for genes with dysregulated expression upon exposure to 1.2 mmol dacarbazine in BRO and SK‐MEL‐2 melanoma cells. Plots were generated using the PANTHER™ v.16.0.

A total of 310 transcripts with altered expression were identified in SK‐MEL‐2 cells versus 8011in BRO cells upon 1.2 mmol dacarbazine treatment (Figure 3A–C). The upregulated genes following dacarbazine treatment in SK‐MEL‐2 cells were associated with cell cycle regulation and quiescence (Caveolin 1 (*CAV*) and Cyclin Dependent Kinase Inhibitor 1A (*CDKNA1*)), extracellular matrix remodeling (Netrin 4 (*NTN4*)), unfolded protein response (Glutathione‐Specific Gamma‐Glutamylcyclotransferase 1 (*CHAC1*)), DNA damage (Tumor Protein P53 Inducible Nuclear Protein 1 (*TP53IMP1*)) and cellular adhesion (Calmegin (*CLGN*)). The downregulated genes were associated with apoptosis (Interferon Alpha Inducible Protein 6 (*IFI6*)) and cell cycle control regulation (Apolipoprotein B MRNA Editing Enzyme Catalytic Subunit 3B (*APOBEC3B*), Kinesin Family Member 20A (*KIF20A*) and Securin (*PTTG1*)). In addition, two genes specific for neuronal cell origin were found among the top downregulated genes following dacarbazine treatment: Protein Cornichon Homolog 2 (*CNIH2*) and Vestigial Like Family Member 1 (*VGLL1*).By contrast, the decrease in G_0_‐positive BRO cells upon dacarbazine treatment was associated with upregulation of apoptosis‐related and gap‐junctions mediated Phoenixin (*PNX2*) and MT‐RNR2 Like 10 (*MTRNR2L10*). GO functional enrichment analysis revealed altered biological processes and classes of proteins in BRO and SK‐Mel‐2 under dacarbazine treatment which are presented in Figure 3F.

Next, the present study focused on those signaling pathways of which the differentially expressed genes were components.

Kyoto Encyclopedia of Genes and Genomes (KEGG) pathway enrichment analysis was performed for the up and downregulated differentially expressed genes. The top dysregulated genes in BRO and SK‐MEL‐2 cells were components of the ‘VEGFA‐VEGFR2’ signaling pathway. Among the dysregulated pathways, ‘Cell cycle’, ‘Focal adhesion’, ‘miRNA Regulation of DNA Damage Response’ consisted of top gene numbers altered upon dacarbazine treatment of BRO and SK‐MEL‐2 cells (Figure 3D).

### 3.5 RT‐qPCR of altered transcripts following dacarbazine treatment of SK‐MEL‐2 and BRO melanoma cells

The expression of several genes was validated by RT‐qPCR, including *CDK4*, *RBL1*, *AURKA*, *TP53* and *MGMT*. *CDK4* expression levels were diminished in BRO cells according to the results of microarray and RT‐qPCR, in SK‐MEL‐2 cells. The *CDK4* levels were decreased according to RT‐qPCR, whereas microarray analysis did not reveal alterations in expression levels. *RBL1* was found to be decreased in BRO and SK‐MEL‐2 cells both by microarray and RT‐qPCR analyses. Decreased *TP53* expression levels were determined in BRO cells both by microarray analysis RT‐qPCR, whereas SK‐MEL‐2 cells were characterized by increased *TP53* expression in accordance with RT‐qPCR. Decreased *AURKA* expression levels were determined by microarray analysis and RT‐qPCR real in SK‐MEL‐2 and in BRO cells. *MGMT* expression was not detected in BRO cells by RT‐qPCR, while it was determined to be not altered by microarray analysis. MGMT expression was determined as not altered in SK‐MEL‐2 cells both by RT‐qPCR and microarray analyses (Figure 3Е). In total, GO functional enrichment analysis revealed altered biological processes and classes of proteins in BRO and SK‐Mel‐2 under dacarbazine treatment which are presented in Figure 3F.

### 3.6 Cell adhesion

**FIGURE 4:**
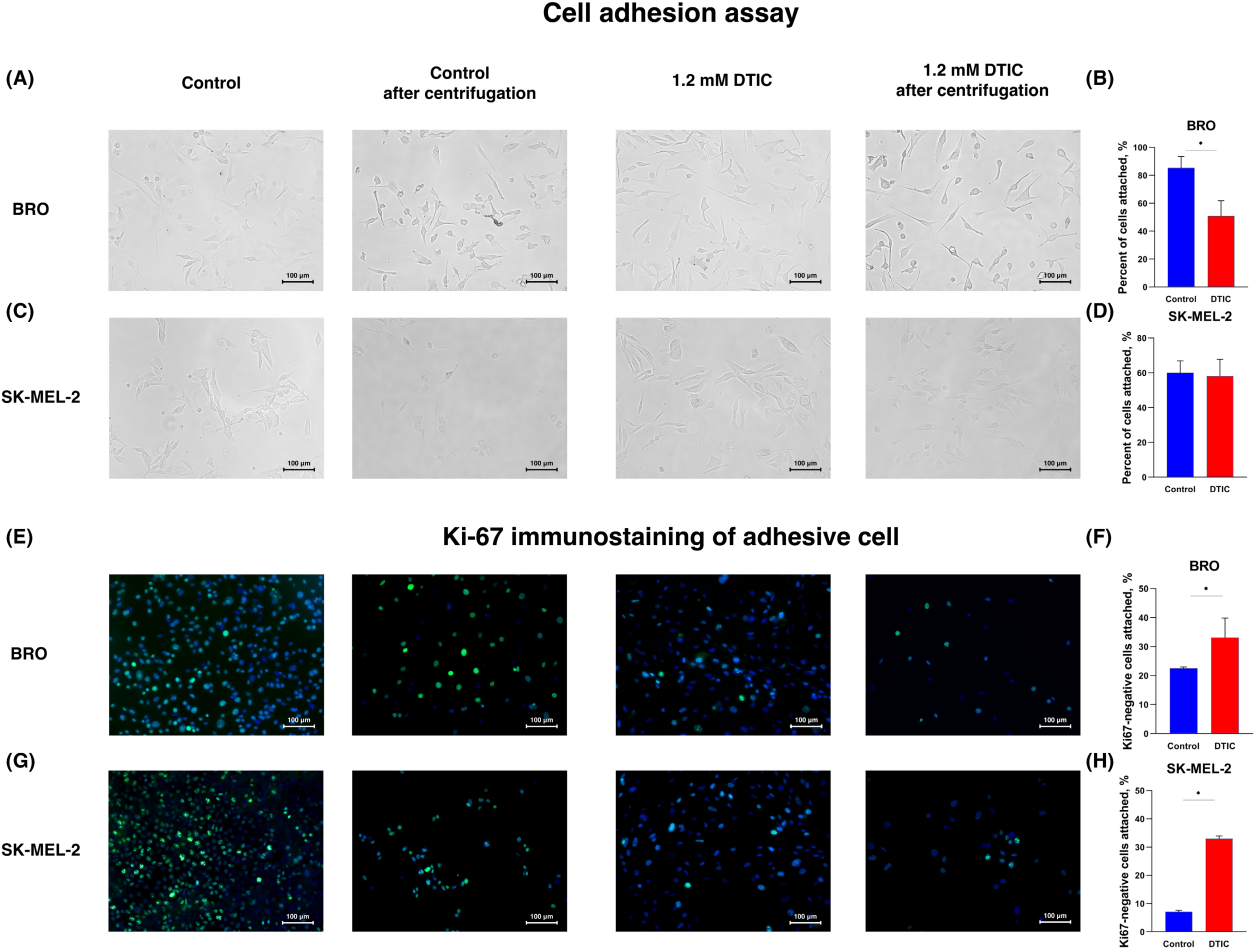
Cell adhesion assay. Representative images of adherent (A) BRO and (C) SK‐MEL‐2 melanoma cells. Quantification of the percentage of adherent (B) BRO and (D) SK‐MEL‐2 cells. Ki‐67 immunostaining of BRO (E) and SK‐MEL‐2 (G) cells after performing cell adhesion assay. Quantification of the percentage of adherent Ki‐67‐negatively stained (F) BRO and (H) SK‐MEL‐2 cells The results are presented as the mean ± standard error of the mean. *p < 0.05 versus control by Mann–Whitney *U* test for unpaired samples.

Since focal adhesion was one of the dysregulated pathways, and several genes associated with cell adhesion were found as altered in accordance with transcriptomic analysis, a cell adhesion assay was performed in melanoma cells following treatment with dacarbazine. Determined a decrease in the adhesive capacities of 1.2 mmol dacarbazine‐treated BRO cells (Figure 4A,B), whereas 1.2 mmol dacarbazine did not alter the adhesion of SK‐MEL‐2 cells (Figure 4C,D). However, when the adhesive features of G_0_‐positive cells were investigated, it was found that both BRO and SK‐MEL‐2 cells showed an increased percentage of G_0_‐positive cells among the adhesive cells (Figure 4E–H).

## 4 DISCUSSION

Melanoma remains the cancer type responsible for the majority of mortalities caused by skin malignancies.^37^ Novel therapeutic approaches, which are based on blocking signaling pathways or inhibiting immune checkpoints, are characterized by the increased overall response and survival rates compared with that caused by chemotherapeutic agents.^38^ However, melanoma cells are highly refractory to any agents used.^39^ Dacarbazine treatment of patients with melanoma is characterized by low response rates with a prevalence of partial rather than complete response.^40^ Several mechanisms were proposed to underly the weak treatment effect of dacarbazine, although clear mechanisms of melanoma chemoresistance remain to be established. It has previously been shown that taxanes induce G_2_/M arrest followed by apoptosis, whereas G_1_‐arrested cells are more resistant to apoptosis.^41^ Similarly, G_1_‐arrested melanoma cells were resistant to the alkylating agent temozolamide.^22^ To further unveil the mechanisms of cell cycle‐dependent resistance, the present study investigated the cell cycle distribution of dacarbazine‐treated melanoma cells, determined the percentage of G_0_‐positive cells and conducted transcriptomic analysis to identify a specific transcription profile of melanoma cells associated with dacarbazine chemoresistance.

Based on PI staining, it was identified that dacarbazine induced cell cycle alterations, which were presented as a G_0_/G_1_ decrease and a G_2_/M increase in SK‐MEL‐2 cells, whereas BRO melanoma cells did not show alterations in cell cycle dynamics. Additional Ki‐67 immunostaining of cells prior to flow cytometry revealed that BRO and SK‐MEL‐2 cells were characterized by an increased percentage of Ki‐67 negative, G_0_‐positive cells. Thus, G_0_‐positive cells percentage alterations were observed upon dacarbazine treatment.

Previously, it was determined that another alkylating agent, temozolomide, induced p53‐dependent G_2_/M cell cycle arrest.^26^ However, the present study observed a G_2_/M weak increase combined with G_0_‐positive cells alterations in SK‐MEL‐2. The G_0_‐positive, Ki‐67 negative cancer cell fraction refers to slow‐cycling cells that originally may represent quiescent cells, senescent cells or both. It is conventionally accepted that quiescent cells represent reversible proliferative arrest, whereas senescent cells irreversibly exited from the cell cycle, although recent studies also consider senescence as a reversible process.^16^, ^42^ Increases in G_0_‐positive cells could be induced by various anticancer agents.^43^ Previous studies have shown that low doses or short periods of treatment with another chemotherapeutic agent, Adriamycin, induced senescence and apoptosis in breast cancer cells, whereas a third type of cell was not either senescent or apoptotic.^44^ By contrast, chemotherapy may induce a reversible effect: the exit of cancer cells from the quiescent to the proliferative state.^45^ Furthermore, recent studies have shown that, in certain cases, the difference between quiescent and senescent cells could be not clear, or the transition between these two cell states could be possible as senescent cells re‐entry the cell cycle.^46^ The differentiation between senescent and quiescent cells within a population of G_0_‐positive cells is challenging, since quiescent cells are not characterized by a specific marker, and both quiescent and senescent cells may share common signaling pathways. However, the percentage of senescent cells in the current study did not exceed 1% in both cell populations studied, and increased by 2‐fold in SK‐MEL‐2 cells upon dacarbazine treatment, which was in agreement with previously published data.^42^

The DNA repair protein O‐6‐methylguanine‐DNA methyltransferase was shown to counteract the alkylating effect of dacarbazine.^47^ The alkylating agent temozolomide has been shown to induce senescence in melanoma cells expressing MGMT.^48^ Since MGMT activity is considered to be one of the key factors affecting cell resistance to alkylating agents, its expression was determined in melanoma cells in the present study. The current results revealed an absence of MGMT and p53 expression in BRO melanoma cells, opposite to the findings in SK‐MEL‐2 cells. Thus, MGMT expression was associated with both senescence and quiescence induction in SK‐MEL‐2 melanoma cells. It was previously shown that MGMT‐expressing melanoma cells could be resistant to p53‐mediated apoptosis.^49^ BRO melanoma cells did not express MGMT; therefore, their resistance to alkylating agents could be associated with other mechanisms.

Next, the present study analyzed the transcriptomic pattern of the two aforementioned melanoma cell lines, which showed a trend in the increase of the percentage of G_0_‐positive cells following dacarbazine treatment. The induction of G_0_‐positive cells in SK‐MEL‐2 cells was associated with dysregulation of the ‘Cell cycle’, ‘VEGFA‐VEGFR2’ and ‘p53 transcriptional gene network’ signaling pathways, where the number of genes upregulated was increased following dacarbazine treatment. However, ‘VEGFA‐VEGFR2’ signaling could be referred to as senescence and SASP,^50^ even though the SASP expression pattern genes Interleukin 6 (*IL‐*6), C‐X‐C Motif Chemokine Ligand 8 (*CXCL8*), Interleukin 1 Alpha (*IL‐1*α), Interleukin 1 Beta (*IL‐1β*), Secreted Phosphoprotein 1 (*SPP1*), Matrix Metallopeptidase 3 (*MMP3*), Matrix Metallopeptidase 2 (*MMP2*), C‐C Motif Chemokine Ligand 2 (*CCL2*), C‐X‐C Motif Chemokine Ligand 1 (*CXCL1*), C‐X‐C Motif Chemokine Ligand 2 (*CXCL2*) and C‐X‐C Motif Chemokine Ligand 5 (*CXCL5*)^51^, ^52^ were not altered in SK‐MEL‐2 or BRO cells upon dacarbazine treatment. However, VEGF produced by tumor cells was shown to stimulate focal adhesion kinase‐mediated vascular permeability in lungs, thus favoring tumor cells homing in lungs,^53^ which is in the line with the present observation of increased G_0_‐positive cells percentage; however, further studies are necessary to demonstrate a potential association between these two events. Among the classical senescence markers CNKN2A, CDKN1A and lamin B1,^54^ CDKN1A only had a 6‐fold increase in SK‐MEL‐2 cells and 2.2‐fold increase in BRO cells following dacarbazine treatment. CDKN1A expression was shown to be dysregulated both in quiescent and senescent cells.^55^ To identify more specific alterations associated with a quiescent melanoma cells phenotype, based on transcriptomic data, genes were selected that were dysregulated both in BRO and in SK‐MEL‐2 cells. Signaling pathways associated with DNA repair and VEGFA were found as dysregulated.

KEGG analysis demonstrated that various genes were significantly enriched in ‘Focal Adhesion’ signaling pathway dysregulation. BRO melanoma cells, where G_0_‐positive cells increased following dacarbazine treatment, were characterized by a similar signaling pathways pattern dysregulation, where the ‘VEGFA‐VEGFR2’ signaling pathway was the most altered, with 118 genes downregulated upon dacarbazine treatment and 6 upregulated. A similar tendency was observed in the ‘Focal Adhesion’ signaling pathway, where 54genes were dysregulated upon dacarbazine treatment. Focal adhesion molecules are implicated in senescence, although their role is not clear. Caveolin‐1 was shown to mediate the hyperadhesive phenotype of senescent cells, which was characterized by focal adhesion kinase activation and diminished cell motility, whereas integrin endocytosis inhibition resulted in reactive oxygen species elevation, thus activating CDKN2A‐mediated senescence in normal human fibroblasts.^56^ The present study determined that BRO cells exhibited decreased adhesive capacities under dacarbazine treatment. Among the G_0_‐positive cells, an increase in cells with elevated adhesive capacities was observed. Senescent cells were found to express adhesive molecules, and the adhesion phenotype of cancer cells has been reported to be strongly associated with their metastatic capacities.^57^ Quiescent cancer cells residing in pre‐metastatic niches need selective communications with the extracellular matrix, which can exert an anchorage and protective function to favor the survival of quiescent cancer cells.^58^ Focal adhesion kinase‐integrin β1 signaling regulates CDKN1A and CDKN1B expression in disseminated cancer cells, and is essential In the present study, human melanoma cells treated with intermediate doses of dacarbazine showed similar alterations of cell distributions in different cell cycle phases. Although dacarbazine has limited therapeutic options, other anticancer agents may induce a similar alteration in cancer cells as our group previously demonstrated with vemurafenib.^59^ Thus, a fraction of cells reversibly enters G_0_, followed by important alterations in the transcriptional profile, where, in addition to apoptosis, DNA damage, cell‐cycle dysregulated molecules and alteration of cell adhesion were observed (Abstract Figure).

However, the present study has several limitations as the results obtained have not yet been verified in a deep functional study. Besides, transition to G_0_ could be observed additionally by the use of genetically encoded fluorescent indicators. Nevertheless, we believe that the metastatic potential of cancer cells entering to G_0_ phase of cell cycle after a treatment by chemotherapeutic agent, as well as adhesive phenotype of the aforementioned cells, requires further study for diagnostic, prognostic and therapeutic purposes.

## AUTHOR CONTRIBUTIONS

**Alexandra R. Esimbekova** Formal analysis (equal); investigation (equal); methodology (equal); writing – original draft (equal). **Nadezhda V. Palkina** Conceptualization (equal); formal analysis (equal); investigation (equal); methodology (equal); writing – original draft (equal); writing – review and editing (equal). **Ivan S. Zinchenko** Investigation (equal); methodology (equal); validation (equal). **Vasiliy D. Belenyuk** Formal analysis (equal); methodology (equal). **Andrey A. Savchenko** Formal analysis (equal); methodology (equal); resources (equal). **Ekaterina Yu Sergeeva** Formal analysis (supporting); methodology (supporting). **Tatiana G. Ruksha** Conceptualization (lead); data curation (lead); funding acquisition (lead); methodology (lead); project administration (lead); resources (lead); supervision (lead); writing – original draft (lead); writing – review and editing (equal).

## FUNDING INFORMATION

The study was supported by the Russian Science Foundation (No. 19‐15‐00110, https://rscf.ru/project/19‐15‐00110/).

## CONFLICT OF INTEREST

Authors declare no conflict of interest.

## ETHICS APPROVAL

This study was approved by the Local Ethics Committee of the Krasnoyarsk State Medical University (approval no 101; date issued October 31, 2020).

## Data Availability

Data generated during the study are subject to a data sharing mandate and available in a public repository Array Express Archive E‐MTAB‐11399 (https://www.ebi.ac.uk/arrayexpress/experiments/E‐MTAB‐11399).

## References

[1] Dulgar O , Kutuk T , Eroglu Z . Mechanisms of resistance to BRAF‐targeted melanoma therapies. Am J Clin Dermatol. 2021;22:1‐10. doi:10.1007/s40257-020-00572-6 33368052

[2] Goldinger SM , Buder‐Bakhaya K , Lo SN , et al. Chemotherapy after immune checkpoint inhibitor failure in metastatic melanoma: a retrospective multicentre analysis. Eur J Cancer (Oxford, England: 1990). 2022. doi:10.1016/j.ejca.2021.11.022 34952480

[3] Carlino MS , Larkin J , Long GV . Immune checkpoint inhibitors in melanoma. Lancet (London, England). 2021;398:1002‐1014. doi:10.1016/S0140-6736(21)01206-X 34509219

[4] Arozarena I , Wellbrock C . Phenotype plasticity as enabler of melanoma progression and therapy resistance. Nat Rev Cancer. 2019;19:377‐391. doi:10.1038/s41568-019-0154-4 31209265

[5] Antonica F , Santomaso L , Pernici D , et al. A slow‐cycling/quiescent cells subpopulation is involved in glioma invasiveness. Nat Commun. 2022;13:4767. doi:10.1038/s41467-022-32448-0 35970913 PMC9378633

[6] Perego M , Maurer M , Wang JX , et al. A slow‐cycling subpopulation of melanoma cells with highly invasive properties. Oncogene. 2018;37:302‐312. doi:10.1038/onc.2017.341 28925403 PMC5799768

[7] Capparelli C , Purwin TJ , Glasheen M , et al. Targeting SOX10‐deficient cells to reduce the dormant‐invasive phenotype state in melanoma. Nat Commun. 2022;13:1381. doi:10.1038/s41467-022-28801-y 35296667 PMC8927161

[8] Carter SK , Friedman MA . 5‐(3,3‐dimethyl‐l‐triazeno)‐imidazole‐4‐carboxamide (DTIC, DIC, NSC‐45388)‐‐a new antitumor agent with activity against malignant melanoma. Eur J Cancer. 1972. doi:10.1016/0014-2964(72)90087-4 4552317

[9] Huber H , Grünewald K . Dacarbazin (DTIC) in der Therapie maligner Erkrankungen. Eine Ubersicht [Dacarbacine (DTIC) in the therapy of a malignant disease. A review (author's transl)]. Wien Klin Wochenschr. 1978;90(24):861‐864. 369151

[10] Kan T , Takahagi S , Kawai M , Matsubara D , Tanaka A , Hide M . Rechallenge of programmed cell death 1 inhibitor after an interval with dacarbazine treatment may be effective for advanced malignant melanoma. J Dermatol. 2020;47:907‐910. doi:10.1111/1346-8138.15408 32515012

[11] Fuchs RP , Isogawa A , Paulo JA , et al. Crosstalk between repair pathways elicits double‐strand breaks in alkylated DNA and implications for the action of temozolomide. Elife. 2021;10. doi:10.7554/eLife.69544 PMC8289412 34236314

[12] Baran K , Yang M , Dillon CP , Samson LL , Green DR . The proline rich domain of p53 is dispensable for MGMT‐dependent DNA repair and cell survival following alkylation damage. Cell Death Differ. 2017;24:1925‐1936. doi:10.1038/cdd.2017.116 28753207 PMC5635218

[13] Qi Z , Tan H . Association between MGMT status and response to alkylating agents in patients with neuroendocrine neoplasms: a systematic review and meta‐analysis. Biosci Rep. 2020;40. doi:10.1042/BSR20194127 PMC7098124 32141507

[14] Damen M , van Rheenen J , Scheele C . Targeting dormant tumor cells to prevent cancer recurrence. FEBS J. 2021;288:6286‐6303. doi:10.1111/febs.15626 33190412

[15] Goddard ET , Bozic I , Riddell SR , Ghajar CM . Dormant tumour cells, their niches and the influence of immunity. Nat Cell Biol. 2018;20:1240‐1249. doi:10.1038/s41556-018-0214-0 30361702

[16] Risson E , Nobre AR , Maguer‐Satta V , Aguirre‐Ghiso JA . The current paradigm and challenges ahead for the dormancy of disseminated tumor cells. Nat Cancer. 2020;1:672‐680. doi:10.1038/s43018-020-0088-5 33681821 PMC7929485

[17] Lee S , Schmitt CA . The dynamic nature of senescence in cancer. Nat Cell Biol. 2019;21:94‐101. doi:10.1038/s41556-018-0249-2 30602768

[18] Paul R , Dorsey JF , Fan Y . Cell plasticity, senescence, and quiescence in cancer stem cells: biological and therapeutic implications. Pharmacol Ther. 2022;231:107985. doi:10.1016/j.pharmthera.2021.107985 34480963 PMC8844041

[19] Ruksha TG . MicroRNAs' control of cancer cell dormancy. Cell Div. 2019;14:11. doi:10.1186/s13008-019-0054-8 31624492 PMC6785928

[20] Ohtani N . The roles and mechanisms of senescence‐associated secretory phenotype (SASP): can it be controlled by senolysis? Inflammation and Regeneration. 2022;42. doi:10.1186/s41232-022-00197-8 PMC8976373 35365245

[21] Pommier A , Anaparthy N , Memos N , et al. Unresolved endoplasmic reticulum stress engenders immune‐resistant, latent pancreatic cancer metastases. Science (New York, NY). 2018. doi:10.1126/science.aao4908 PMC6547380 29773669

[22] Beaumont KA , Hill DS , Daignault SM , et al. Cell cycle phase‐specific drug resistance as an escape mechanism of melanoma cells. J Invest Dermatol. 2016;136:1479‐1489. doi:10.1016/j.jid.2016.02.805 26970356

[23] Eigner K , Filik Y , Mark F , et al. The unfolded protein response impacts melanoma progression by enhancing FGF expression and can be antagonized by a chemical chaperone. Sci Rep. 2017;7:17498. doi:10.1038/s41598-017-17888-9 29235576 PMC5727496

[24] Wenzel AT , Champa D , Venkatesh H , et al. Single‐cell characterization of step‐wise acquisition of carboplatin resistance in ovarian cancer. NPJ Syst Biol Appl. 2022;8(1):20. doi:10.1038/s41540-022-00230-z 35715421 PMC9206019

[25] Basu S , Dong Y , Kumar R , Jeter C , Tang DG . Slow‐cycling (dormant) cancer cells in therapy resistance, cancer relapse and metastasis. Semin Cancer Biol. 2022;78:90‐103. doi:10.1016/j.semcancer.2021.04.021 33979674 PMC8576068

[26] Mhaidat NM , Zhang XD , Allen J , Avery‐Kiejda KA , Scott RJ , Hersey P . Temozolomide induces senescence but not apoptosis in human melanoma cells. Br J Cancer. 2007;97:1225‐1233. doi:10.1038/sj.bjc.6604017 17968428 PMC2360470

[27] Sun X , Shi B , Zheng H , et al. Senescence‐associated secretory factors induced by cisplatin in melanoma cells promote non‐senescent melanoma cell growth through activation of the ERK1/2‐RSK1 pathway. Cell Death Dis. 2018;9:260. doi:10.1038/s41419-018-0303-9 29449532 PMC5833767

[28] Kim KH , Sederstrom JM . Assaying cell cycle status using flow cytometry. Curr Protoc Mol Biol. 2015;111:28.6.1‐28.6.11. doi:10.1002/0471142727.mb2806s111 PMC4516267 26131851

[29] De Backer J , Maric D , Bosman M , Dewilde S , Hoogewijs D . A reliable set of reference genes to normalize oxygen‐dependent cytoglobin gene expression levels in melanoma. Sci Rep. 2021;11:10879. doi:10.1038/s41598-021-90284-6 34035373 PMC8149659

[30] Jiffry J , Thavornwatanayong T , Rao D , et al. Oncolytic Reovirus (pelareorep) induces autophagy in KRAS‐mutated colorectal cancer. Clin Cancer Res. 2021;27:865‐876. doi:10.1158/1078-0432.CCR-20-2385 33168658 PMC8130598

[31] Pan Q , Qiu WY , Huo YN , Yao YF , Lou MF . Low levels of hydrogen peroxide stimulate corneal epithelial cell adhesion, migration, and wound healing. Invest Ophthalmol Vis Sci. 2011;52:1723‐1734. doi:10.1167/iovs.10-5866 21087961 PMC3101689

[32] Tanaka R , Goshima F , Esaki S , et al. The efficacy of combination therapy with oncolytic herpes simplex virus HF10 and dacarbazine in a mouse melanoma model. Am J Cancer Res. 2017;7(8):1693‐1703. 28861325 PMC5574941

[33] Reid JM , Kuffel MJ , Miller JK , Rios R , Ames MM . Metabolic activation of Dacarbazine by human cytochromes P450: the role of CYP1A1, CYP1A2, and CYP2E11. Clin Cancer Res. 1999;5(8):2192‐2197. 10473105

[34] Katiyar SK , Matsui MS , Mukhtar H . Ultraviolet‐B exposure of human skin induces cytochromes P450 1A1 and 1B1. J Invest Dermatol. 2000;114:328‐333. doi:10.1046/j.1523-1747.2000.00876.x 10651994

[35] Tyumentseva A , Averchuk A , Palkina N , et al. Transcriptomic profiling revealed Plexin A2 downregulation with migration and invasion alteration in dacarbazine‐treated primary melanoma cells. Front Oncol. 2021;11. doi:10.3389/fonc.2021.732501 PMC8677675 34926249

[36] Gorgoulis V , Adams PD , Alimonti A , et al. Cellular senescence: defining a path forward. Cell. 2019;179:813‐827. doi:10.1016/j.cell.2019.10.005 31675495

[37] Garbe C , Keim U , Gandini S , et al. Epidemiology of cutaneous melanoma and keratinocyte cancer in white populations 1943‐2036. Eur J Cancer (Oxford, England: 1990). 2021. doi:10.1016/j.ejca.2021.04.029 34062483

[38] Nguyen K , Hignett E , Khachemoune A . Current and emerging treatment options for metastatic melanoma: a focused review. Dermatol Online J. 2020;26(7):13030/qt24g3k7z5. 32898395

[39] Kozar I , Margue C , Rothengatter S , Haan C , Kreis S . Many ways to resistance: how melanoma cells evade targeted therapies. Biochim Biophys Acta. 2019. doi:10.1016/j.bbcan.2019.02.002 30776401

[40] Pasquali S , Hadjinicolaou AV , Chiarion Sileni V , Rossi CR , Mocellin S . Systemic treatments for metastatic cutaneous melanoma. Cochrane Database Syst Rev. 2018;2020:CD011123. doi:10.1002/14651858.CD011123.pub2 PMC6491081 29405038

[41] Abal M , Andreu JM , Barasoain I . Taxanes: microtubule and centrosome targets, and cell cycle dependent mechanisms of action. Curr Cancer Drug Targets. 2003;3:193‐203. doi:10.2174/1568009033481967 12769688

[42] Saleh T , Gewirtz DA . Considering therapy‐induced senescence as a mechanism of tumour dormancy contributing to disease recurrence. Br J Cancer. 2022;126:1363‐1365. doi:10.1038/s41416-022-01787-6 35304605 PMC9091207

[43] Paffenholz SV , Salvagno C , Ho YJ , et al. Senescence induction dictates response to chemo‐ and immunotherapy in preclinical models of ovarian cancer. Proc Natl Acad Sci USA. 2022;119. doi:10.1073/pnas.2117754119 PMC8812522 35082152

[44] Song YS , Lee BY , Hwang ES . Dinstinct ROS and biochemical profiles in cells undergoing DNA damage‐induced senescence and apoptosis. Mech Ageing Dev. 2005;126:580‐590. doi:10.1016/j.mad.2004.11.008 15811427

[45] Xie XP , Laks DR , Sun D , et al. Quiescent human glioblastoma cancer stem cells drive tumor initiation, expansion, and recurrence following chemotherapy. Dev Cell. 2022;57:32‐46.e8. doi:10.1016/j.devcel.2021.12.007 35016005 PMC8820651

[46] Yang C , Tian C , Hoffman TE , Jacobsen NK , Spencer SL . Melanoma subpopulations that rapidly escape MAPK pathway inhibition incur DNA damage and rely on stress signalling. Nat Commun. 2021;12:1747. doi:10.1038/s41467-021-21549-x 33741929 PMC7979728

[47] Azimi A , Pernemalm M , Frostvik Stolt M , et al. Proteomics analysis of melanoma metastases: association between S100A13 expression and chemotherapy resistance. Br J Cancer. 2014;110:2489‐2495. doi:10.1038/bjc.2014.169 24722184 PMC4021518

[48] Erice O , Smith MP , White R , et al. MGMT expression predicts PARP‐mediated resistance to Temozolomide. Mol Cancer Ther. 2015;14:1236‐1246. doi:10.1158/1535-7163.MCT-14-0810 25777962

[49] Barckhausen C , Roos WP , Naumann SC , Kaina B . Malignant melanoma cells acquire resistance to DNA interstrand cross‐linking chemotherapeutics by p53‐triggered upregulation of DDB2/XPC‐mediated DNA repair. Oncogene. 2014;33:1964‐1974. doi:10.1038/onc.2013.141 23604128

[50] Sharpless NE , Sherr CJ . Forging a signature of in vivo senescence. Nat Rev Cancer. 2015;15:397‐408. doi:10.1038/nrc3960 26105537

[51] Funck F , Pahl J , Kyjacova L , et al. Human innate immune cell crosstalk induces melanoma cell senescence. Onco Targets Ther. 2020;9. doi:10.1080/2162402X.2020.1808424 PMC7470184 32939325

[52] Kyjacova L , Saup R , Rönsch K , et al. IER2‐induced senescence drives melanoma invasion through osteopontin. Oncogene. 2021;40:6494‐6512. doi:10.1038/s41388-021-02027-6 34611309 PMC8616759

[53] Hiratsuka S , Goel S , Kamoun WS , et al. Endothelial focal adhesion kinase mediates cancer cell homing to discrete regions of the lungs via E‐selectin up‐regulation. Proc Natl Acad Sci USA. 2011;108:3725‐3730. doi:10.1073/pnas.1100446108 21321210 PMC3048115

[54] Hernandez‐Segura A , de Jong TV , Melov S , Guryev V , Campisi J , Demaria M . Unmasking transcriptional heterogeneity in senescent cells. Curr Biol. 2017;27:2652‐2660.e4. doi:10.1016/j.cub.2017.07.033 28844647 PMC5788810

[55] Overton KW , Spencer SL , Noderer WL , Meyer T , Wang CL . Basal p21 controls population heterogeneity in cycling and quiescent cell cycle states. Proc Natl Acad Sci USA. 2014;111:E4386‐E4393. doi:10.1073/pnas.1409797111 25267623 PMC4205626

[56] Shin EY , Park JH , You ST , et al. Integrin‐mediated adhesions in regulation of cellular senescence. Sci Adv. 2020;6:eaay3909. doi:10.1126/sciadv.aay3909 32494696 PMC7202880

[57] Yeoman B , Shatkin G , Beri P , Banisadr A , Katira P , Engler AJ . Adhesion strength and contractility enable metastatic cells to become adurotactic. Cell Rep. 2021;34:108816. doi:10.1016/j.celrep.2021.108816 33691109 PMC7997775

[58] Celià‐Terrassa T , Kang Y . Metastatic niche functions and therapeutic opportunities. Nat Cell Biol. 2018;20:868‐877. doi:10.1038/s41556-018-0145-9 30050120

[59] Nikolaeva ED , Dubovtseva IY , Belonogov RN , et al. Vemurafenib‐induced increase in Ki‐67‐negative cells in BRAF‐negative melanoma. Cell Tiss. Biol. 2021. doi:10.1134/S1990519X2103007X

